# Antiphospholipid Antibodies and Infection: *Non Nova Sed Nove*

**DOI:** 10.3389/fimmu.2021.687534

**Published:** 2021-06-16

**Authors:** Savino Sciascia, Massimo Radin, Mario Bazzan, Barbara Montaruli, Domenico Cosseddu, Claudio Norbiato, Maria Tiziana Bertero, Renato Carignola, Beatrice Bacco, Silvia Gallo Cassarino, Dario Roccatello

**Affiliations:** ^1^ Center of Research of Immunopathology and Rare Diseases, Coordinating Center of Piemonte and Valle d’Aosta Network for Rare Diseases, Department of Clinical and Biological Sciences, University of Turin and S. Giovanni Bosco Hospital, Turin, Italy; ^2^ Nephrology and Dialysis Unit, S. Giovanni Bosco Hospital and University of Turin, Turin, Italy; ^3^ Hematology Division, S. Giovanni Bosco Hospital, Turin, Italy; ^4^ AO Ordine Mauriziano, Turin, Italy; ^5^ Internal Medicine, San Luigi Gonzaga Hospital Orbassano, Turin, Italy

**Keywords:** antiphospholipid antibodies, infection, COVID-19, antiphospholipid antibody syndrome, thrombosis

## Abstract

The clinical significance of antiphospholipid antibodies (aPL) in the context of infections has attracted attention since their first discovery in patients with syphilis. In fact, the recognition of aPL in patients with infections has been described in parallel to the understating of the syndrome. Since the first description of aPL-positive tests in three patients with COVID-19 diagnosed in January 2020 in Wuhan, China, a large number of studies took part in the ongoing debate on SARS-2-Cov 2 induced coagulopathy, and many following reports speculated a potential role for aPL. In order to get further insights on the effective role of detectable aPL in the pro-thrombotic status observed in COVID-19 patients, we performed an observational age-sex controlled study to compare the aPL profile of hospitalized patients with COVID with those observed in a) patients with thrombotic APS and b) patients with cultural/serologically-proved infections. Our data showed positive aPL testing in about half of the patients (53%) with COVID-19 and patients with other viral/bacterial infections (49%). However, aPL profile was different when comparing patients with overt APS and patients with aPL detected in the contest of infections. Caution is therefore required in the interpretation and generalization of the role of aPL s in the management of patients with COVID-19. Before introducing aPL testing as a part of the routine testing in patients with COVID-19, larger well-designed clinical studies are required. While the pro-thrombotic status in patients with COVID-19 is now unquestionable, different mechanisms other than aPL should be further investigated.

## Introduction

The antiphospholipid syndrome (APS) is a systemic autoimmune condition characterized by the persistent elevation of antiphospholipid antibodies (aPL), such as anticardiolipin antibodies (aCL), lupus anticoagulant (LA), and anti-beta2 Glycoprotein 1 (aβ2GPI), in patients with thromboembolic events and/or pregnancy-related morbidity (1) (**Box 1**). The clinical significance of aPL in the context of infections has attracted attention since their first discovery in patients with syphilis (2). In fact, the recognition of aPL in patients with infections has been described in parallel to the understating of the syndrome (3). Since the global outbreak of the COVID-19 pandemic, a potential relationship between the presence of aPL and the new has been largely debated.

Box 1 **Classification criteria of the Antiphospholipid Syndrome.**

Clinical criteria

- Thrombosis affecting the arteries, veins, or small blood vessels and/or
- Adverse outcomes during pregnancies (three or more spontaneous abortions before 10th week of pregnancy, unexplained fetal deaths at or beyond 10th week of pregnancy, or premature births before 34th week of pregnancy due to severe preeclampsia or eclampsia)

Laboratory criteria (antiphospholipid antibody tests, to be confirmed at 12 weeks)

- Positive lupus anticoagulant test and/or
- Positive anticardiolipin antibody (aCL) IgG and/or IgM and/or
- Positive anti-Beta-2-glycoprotein-I antibody (aβ2GPI) IgG and/or IgM

First, Zhang and co-workers (4) reported a 69-year-old man with COVID-19 diagnosed in January 2020 in Wuhan, China, along with two other critically ill patients with COVID-19 who were also seen in the same intensive care unit. aPL were detected in all three patients. Since then, a large number of studies took part in the ongoing debate on SARS-2-Cov 2–induced coagulopathy, and many following reports speculated a potential role for aPL.

Recently, Zou et al. (5), when testing 172 patients hospitalized with COVID-19 for an extended panel of aPL, found that aPL were present in up to 52% of serum samples using the manufacturer’s threshold and in 30% using a more stringent cut-off (≥40 ELISA-specific units). In detail, among the various aPL antibodies tested, anti-phosphatidylserine/prothrombin (aPS/PT) IgG had the highest prevalence (24%), followed by aCL IgM (23%) and aPS/PT IgM (18%). Forty-one patients (24%) were positive for more than one type of aPL antibody, and 13 (8%) were positive for more than two types of aPL antibody. Fifty-two patients (30%) had at least one moderate- to high-titer aPL antibody. Interestingly, they reported that IgG fractions isolated from patients with COVID-19 and high serum titers for aPS/PT IgG increased thrombus extension in a murine model. While the pro-thrombotic profile of patients with COVID-19 is unquestionable, the specific role of aPL in this setting still requires further considerations.

Do the detectable aPL actively participate in the pro-thrombotic status observed in COVID-19 patients or they represent an epiphenomenon in the context of the infection, as previously described in other settings?

In order to get further insights on the effective role of detectable aPL in the pro-thrombotic status observed in COVID-19 patients, we performed an observational age-sex controlled study to compare the aPL profile of hospitalized patients with COVID with those observed in a) patients with thrombotic APS and b) patients with cultural/serologically-proved infections.

## Methods

We included 261 patients (divided in three age and sex-matched controls groups of 87 patients):

1) Consecutive PCR-confirmed COVID-19–infected patients admitted at the AO Ordine Mauriziano Hospital, Torino, Italy
2) Age- and sex-matched controls with viral and bacterial infections* and no previous history of thrombotic events attending the S. Giovanni Bosco Hospital, Torino, Italy
3) Age and sex-matched patients with APS fulfilling Sidney’s criteria (1) admitted at the S. Giovanni Bosco Hospital, Torino, Italy

The aetiology of infections was: 24 cases of Treponema pallidum, 18 cases of cytomegalovirus, 10 cases of Influenza H3N2, 10 of E. coli, 8 of Streptococcus pneumoniae, 7 of parvovirus B19, 7 of Epstein-Barr virus, and 2 of Pseudomonas aeruginosa (**Table 1**).

**Table 1:** Rate of positive antiphospholipid antibodies’ testing and relative differences between groups.

	Group A Patients with infections(n = 87)	P value (Group A *vs*. B)	Group B COVID-19 Patients(n = 87)	P value (Group B *vs*. C)	Group C Patients with APS (n = 87)	P value (Group B *vs*. C)
**Lupus Anticoagulant positive (n)**	41	0.02	26	<0.0001	76	<0.0001
**β2GPI IgM positive(n)**	17	0.002	4	n.s.	9	0.046
**β2GPI IgM titers (U/mL; mean ± SD)**	25.8 ± 57.1	0.0003	2.7 ± 5.6	n.s.	9.9 ± 28.3	n.s.
**β2GPI IgG positive(n)**	11	n.s.	12	<0.0001	41	<0.0001
**β2GPI IgG titers (U/mL; mean ± SD)**	12 ± 14.6	n.s.	8 ± 8.9	<0.0001	57.9 ± 92.4	<0.0001
**aCL IgM positive(n)**	18	0.0004	4	<0.0001	28	0.0015
**aCL IgM titers (U/mL; mean ± SD)**	19.8 ± 33	n.s.	3.8 ± 5.3	n.s.	17.5 ± 36.8	n.s.
**aCL IgG positive(n)**	11	n.s.	11	0.002	42	0.002
**aCL IgG titers (U/mL; mean ± SD)**	14.7 ± 27.8	n.s.	10.1 ± 16.3	<0.0001	69.3 ± 108.3	<0.0001
**aPS/PT IgM positive (n)**	22	0.0005	7	<0.0001	51	<0.0001
**aPS/PT IgM titers (U/mL; mean ± SD)**	36.1 ± 48.9	<0.0001	10.6 ± 15.7	<0.0001	137 ± 133.5	<0.0001
**aPS/PT IgG positive (n)**	0	n.s.	0	<0.0001	34	<0.0001
**aPS/PT IgG titers (U/mL; mean ± SD)**	9.3 ± 6.2	<0.0001	3.7 ± 4.3	<0.0001	83.8 ± 101.9	<0.0001
**Double Criteria aPL positive (IgG/IgM) (n)**	16	0.043	7	<0.0001	38	<0.0001
**Triple Criteria aPL positive (IgG/IgM) (n)**	7	n.s.	2	<0.0001	35	<0.0001
**Triple Criteria aPL (IgG/IgM) and aPS/PT (IgG/IgM) positive (n)**	3	n.s.	0	<0.0001	30	<0.0001

APS, antiphospholipid Syndrome; aPL, antiphospholipid antibodies; aPS/PT, anti-phosphatidylserine/prothrombin antibodies; aβ2GPI, anti-β2-glycoprotein-I antibodies; aCL, anticardiolipin antibodies; Ig, immunoglobulin; n.s., non significant.

The IgG/IgM isotype for aCL, aβ2GPI and aPS/PT were detected by commercial ELISA (Inova Diagnostics, Inc., San Diego, CA, US).

LA was tested as per the current criteria from the International Society of Thrombosis and Haemostasis (ISTH) Subcommittee on LA-Phospholipid-dependent antibodies (6).

The significance of baseline differences was determined by the chi-squared test, Fisher’s exact test or the unpaired t-test, as appropriate. A two-sided P-value <0.05 was statistically significant. All statistical analyses were performed using SPSS version 26.0 (IBM, Armonk, NY, USA).

## Results

A total of 261 patients were enrolled in this study, 87 patients for each group: patients with COVID-19 infection, matched-controls with infections other than COVID-19 and matched-APS patients. As per classification criteria, all patients with APS were positive for aPL, while a high rate of patients positive for at least one aPL (IgG/IgM) was observed similarly in patients with COVID-19 infection (46; 52.9%) and controls suffering with other infections (43; 49.4%).

PCR-confirmed COVID-19–infected patients were tested for aPL at the time of the admission in the Hospital, 84 patients (96.7%) were treated with low molecular weight heparins. Only one patient developed a deep vein thrombosis during the admission. **Table 1** resumes the rate of positive antiphospholipid antibodies’ testing and relative differences between groups.

When focusing on criteria aPL, as expected, APS patients had significantly higher rates of positive testing for LA when compared with the other groups [87.4% *vs*. 29.9% (COVID-19) and *vs*. 47.1% (infections)] and for IgG isotype of aß2GPI [47.1% vs. 13.8% (COVID-19) and *vs*. 12.6% (infections)] and aCL [48.3% *vs*. 12.6% (COVID-19) and *vs*. 12.6% (infections)]. Also, when looking at multiple criteria aPL positive test (considering IgG and/or IgM isotypes), the APS group had significantly higher rates of double aPL positive testing [43.7% *vs*. 8% (COVID-19) and *vs*. 18.4% (infections)], triple aPL positive testing [40.2% *vs*. 2.2% (COVID-19) and *vs*. 8% (infections)].

The trend was confirmed also when considering aPS/PT testing, as non-criteria aPL. APS patients were the only group with patients that tested positive for aPS/PT IgG (39.1%) and had significantly higher rates of aPS/PT IgM testing [58.6% *vs*. 8% (COVID-19) and *vs*. 25.3% (infections)]. Additionally, 34.5% of APS patients tested positive for both all three criteria aPL and aPS/PT IgG/M (tetra positive patients), when compared to only three patients of the infections group and no patients in the COVID-19 infection group.

The results of the analysis, for both criteria and non-criteria aPL, were also confirmed when analyzing the titers of the aPL tested (**Table 1**).

**Figure 1:**
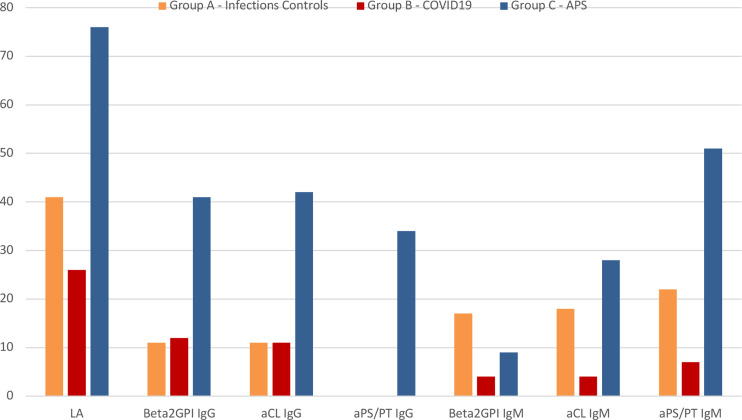
Graphical representation of the rate of antiphospholipid antibodies positive patients between groups. APS, antiphospholipid syndrome; LA, lupus anticoagulant; aPS/PT, anti-phosphatidylserine/prothrombin antibodies; aβ2GPI, anti-β2-glycoprotein-I antibodies; aCL, anticardiolipin antibodies; Ig, immunoglobulin.

**Figure 1** graphically illustrates the different rate of aPL positive testing between groups.

No patients with aPL-positive test reported any thrombotic events (both COVID-19 and infections group). In the COVID-19 group, we observed that no previously positive solid phase aPL test was confirmed when re-testing 12 patients at more than 12 weeks part. Of those, of the five LA-positive tests, only two were confirmed, albeit with a marked reduction of LA potency.

## Discussion

Our data showed positive aPL testing in about half of the patients (53%) with COVID-19 and patients with other viral/bacterial infections (49%). In detail, we found that a positive test for LA can be detected in up to one out of three symptomatic COVID-19 patients when tested according to the ISTH (6). However, the so called triple aPL positivity (concomitant presence of LA, aCL, and aβ2GPI antibodies), the *aPL* profile most strongly associated with a thrombotic event in patients with APS, has been observed in only two patients (2%). More importantly, most of the aCL and aβ2GPI antibodies positive testing was detected at the low-medium titer. Interestingly, in our cohort, no patient with infection (both in the COVID-19 or infection groups) was found positive for aPS/PT IgG.

aPL positivity are known to be detectable during infections, to include viral diseases, such as HIV and hepatitis C. The presence of aPL in these contexts is often transient and almost always non-specific (non-thrombosis-related). While syphilis is notorious for the possible determination of aPL, when considering our cohort, we did not observe a different rate of aPL positive testing between patients with syphilis when compared to other infections. However, it is likely that the sample size limited the feasibility of any additional sub-analysis.

When comparing our data to Zuo et al. (5), some considerations are worth noting. So far, LA-positive testing was the mostly frequently reported among patients with COVID tested for aPL. From this perspective, as a limit to their analysis, they acknowledged that the LA test was not performed given lack of access to fresh plasma samples. When considering solid-phase aPL assays, methods that are in principle insensitive to anticoagulation and other confounding agents, the presence of aPL in patients with COVID-19 was recently reported in a handful of case reports and small cohorts of patients (7, 8). While encouraging, these data are limited and its interpretation remains controversial, with some investigators proposing an important role of aPL in COVID-19 patients (7, 8) while others suggesting no association between aPL and thrombotic events (9, 10). There is no information on the antigen specificity of COVID-19 aPL in comparison with APS antibodies. Such information and a larger study, possibly multicenter, may be instrumental to clarify the real clinical value of these autoantibodies.

Our results support what Borghi et al. suggested (9). In fact, when testing for aPL by both ELISA and chemiluminescence 122 sera of patients with COVID, they (9) found that anti-β2GPI IgG were detectable in about 15%, with IgA and IgM respectively in 6.6 and 9.0% of patients. aCL IgG/IgM was detected in 5.7/6.6% and aPS/PT IgG/IgM were detectable in 2.5% and 9.8%. Critically, no association between thrombosis and aPL was found. Reactivity against domain 1 and 4 to 5 of β2GPI was limited to 3/58 (5.2%) tested sera for each domain and did not correlate with aCL/anti-β2GPI nor with thrombosis.

Some limitations should be acknowledged. Patients enrolled in the control infectious group were chart review-selected by matching cases for sex and age group with 1 year difference allowance. Taking this into account, it was out of the scope of this paper to report on any estimations on specific infection rates or epidemiological inference for this group and to provide evidence on the potential impact of clinical disease on aPL development. Similarly, it was out of the scope of this study to investigate thrombosis rates in patients with COVID-19 infections.

In such a dramatic moment globally, researchers are called to the urgent need for a better understanding of COVID-19–related syndrome. On the other hand, before solid evidence is available, caution is warranted to minimize the risk of unjustified requests for tests to be performed in laboratories that are already overloaded during the ongoing health emergency. Besides, with the current level of evidence, the new detection of aPL in a patient with COVID-19 should not guide the management of the anti-thrombotic therapy that should be based on the available international guidelines.

In conclusion, caution is therefore required in the interpretation and generalization of the role of aPL such as aPS/PT in the management of patients with COVID-19. Before introducing aPL testing as a part of the routine testing in patients with COVID-19, larger well-designed clinical studies are required. While the pro-thrombotic status in patients with COVID-19 is now unquestionable, different mechanisms other than aPL should be further investigated.

## Data Availability Statement

The raw data supporting the conclusions of this article will be made available by the authors, without undue reservation.

## Ethics Statement

The studies involving human participants were reviewed and approved by Comitato Etico Interaziendale Città Di Torino. The patients/participants provided their written informed consent to participate in this study.

## Author Contributions

SS and MB designed the study, analyzed the data, and drafted the manuscript. MR, MB, DC, CN, MTB, RC, BB, SG, and DR participated in case collection, laboratory analysis, and data analysis and criticially participated in the manuscript. All authors contributed to the article and approved the submitted version.

## Funding

MR is funded by a grant from the Italian Ministry of Health (SG-2018-12368028).

## Conflict of Interest

The authors declare that the research was conducted in the absence of any commercial or financial relationships that could be construed as a potential conflict of interest.
